# (*Z*)-*N*-Methyl-2-(5-nitro-2-oxoindolin-3-yl­idene)hydrazinecarbothio­amide

**DOI:** 10.1107/S1600536812001183

**Published:** 2012-03-03

**Authors:** Amna Qasem Ali, Naser Eltaher Eltayeb, Siang Guan Teoh, Abdussalam Salhin, Hoong-Kun Fun

**Affiliations:** a School of Chemical Sciences, Universiti Sains Malaysia, Minden, Penang, Malaysia; b Faculty of Science, Sabha University, Libya; c Department of Chemistry, International University of Africa, Sudan; d X-ray Crystallography Unit, School of Physics, Universiti Sains Malaysia, 11800 USM, Penang, Malaysia

## Abstract

In the title compound, C_10_H_9_N_5_O_3_S, an intra­molecular N—H⋯O hydrogen bond generates an *S*(6) ring motif. In the crystal, mol­ecules are linked *via* N—H⋯S hydrogen bonds into a zigzag chain along the *b* axis. C—H⋯O inter­actions are observed between the chains.

## Related literature

For related structures, see: Qasem Ali *et al.* (2011*a* ▶,*b* ▶); Ferrari *et al.* (2002 ▶); Pervez *et al.* (2010 ▶); Ramzan *et al.* (2010 ▶). For various biological activities of Schiff bases, see: Bhandari *et al.* (2008 ▶); Bhardwaj *et al.* (2010 ▶); Pandeya *et al.* (1999 ▶); Sridhar *et al.* (2002 ▶); Suryavanshi & Pai (2006 ▶). For the cytotoxic and anti­cancer activity of isatin and its derivatives, see: Vine *et al.* (2009 ▶). For graph-set analysis, see: Bernstein *et al.* (1995 ▶).
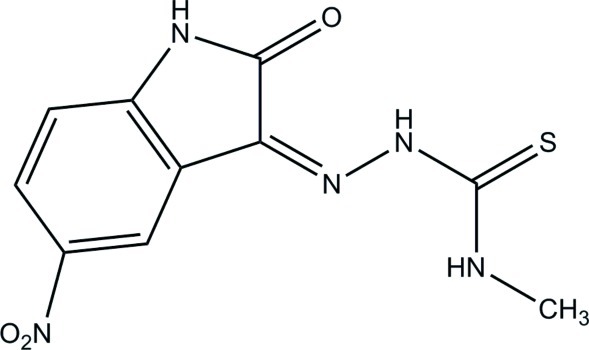


## Experimental

### 

#### Crystal data

C_10_H_9_N_5_O_3_S
*M* *_r_* = 279.28
Monoclinic, 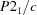

*a* = 4.6316 (4) Å
*b* = 9.3157 (8) Å
*c* = 26.458 (2) Å
β = 94.485 (2)°
*V* = 1138.09 (17) Å^3^
*Z* = 4
Mo *K*α radiation
μ = 0.30 mm^−1^
*T* = 100 K
0.36 × 0.12 × 0.07 mm

#### Data collection

Bruker APEXII CCD diffractometer
Absorption correction: multi-scan (*SADABS*; Bruker, 2005 ▶) *T*_min_ = 0.900, *T*_max_ = 0.979
10734 measured reflections
2710 independent reflections
2177 reflections with *I* > 2σ(*I*)
*R*_int_ = 0.045

#### Refinement

*R*[*F* ^2^ > 2σ(*F* ^2^)] = 0.048
*wR*(*F* ^2^) = 0.102
*S* = 1.10
2710 reflections
185 parameters
H atoms treated by a mixture of independent and constrained refinement
Δρ_max_ = 0.40 e Å^−3^
Δρ_min_ = −0.29 e Å^−3^

### 

Data collection: *APEX2* (Bruker, 2005 ▶); cell refinement: *SAINT* (Bruker, 2005 ▶); data reduction: *SAINT*; program(s) used to solve structure: *SHELXS97* (Sheldrick, 2008 ▶); program(s) used to refine structure: *SHELXL97* (Sheldrick, 2008 ▶); molecular graphics: *SHELXTL* (Sheldrick, 2008 ▶); software used to prepare material for publication: *SHELXTL* and *PLATON* (Spek, 2009 ▶).

## Supplementary Material

Crystal structure: contains datablock(s) I, global. DOI: 10.1107/S1600536812001183/is5048sup1.cif

Structure factors: contains datablock(s) I. DOI: 10.1107/S1600536812001183/is5048Isup2.hkl

Supplementary material file. DOI: 10.1107/S1600536812001183/is5048Isup3.cml

Additional supplementary materials:  crystallographic information; 3D view; checkCIF report

## Figures and Tables

**Table 1:** Hydrogen-bond geometry (Å, °)

*D*—H⋯*A*	*D*—H	H⋯*A*	*D*⋯*A*	*D*—H⋯*A*
N3—H1*N*3⋯O1	0.84 (3)	2.02 (3)	2.697 (2)	137 (3)
N1—H1*N*1⋯S1^i^	0.81 (3)	2.52 (3)	3.320 (2)	171 (3)
C2—H2*A*⋯O1^ii^	0.95	2.39	3.317 (3)	165
C10—H10*A*⋯O2^iii^	0.98	2.56	3.079 (3)	113

Symmetry codes: (i) 
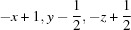
; (ii) 
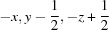
; (iii) 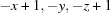.

**Fig. 1.**
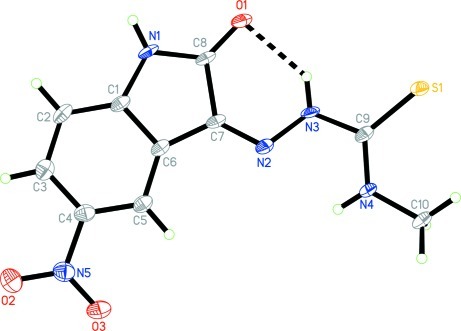
The molecular structure of the title compound, with 50% probability displacement ellipsoids and the atom-numbering scheme.

**Fig. 2.**
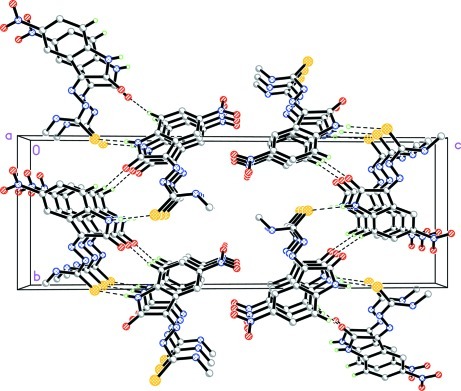
The crystal packing of the title compound viewed down the a axis. Hydrogen bonds are shown as dashed lines.

## supplementary crystallographic information

### Comment

Isatin (2,3-dioxindole) is an endogenous compound identified in humans, and its effect has been studied in a variety of systems. Biological properties of isatin and its derivatives include a range of actions in the brain, offer protection against bacterial (Suryavanshi & Pai, 2006) and antifungal infections and possess anticonvulsant, anti-HIV (Pandeya *et al.*, 1999), anti-depressant and anti-inflammatory activities (Bhandari *et al.*, 2008). Recently, we reported the crystal structure of (*Z*)-2-(5-chloro-2-oxoindolin-3-ylidene)-*N*-phenylhydrazinecarbothioamide (Qasem Ali *et al.*, 2011*a*). In the present paper we describe the single-crystal X-ray diffraction study of title compound, Fig. 1.

In this compound (Fig. 1), the chain N2/N3//C9/S1/N4/C10 connected to the nine-membered 5-nitroindolin-2-one ring system in C7. In this chain, C7—N2—N3—C9 and C10—N4—C9—S1 have torsion angles 177.82 (19) and -0.9 (3)°, respectively. The essentially planar conformation of the molecule is maintained by an intramolecular N3—H1N3···O1 hydrogen bond (Table 1) with a graph-set *S*(6) (Bernstein *et al.*, 1995) In the crystal, molecules are linked *via* an intermolecular N1—H1N1···S1^i^ hydrogen bond into an infinite one-dimensional chain along the *b* axis (Table 1 and Fig. 2). C2—H2A···O1^ii^ and C10—H10A···O2^iii^ hydrogen bonds (Table 1) are also observed between the chains.

### Experimental

The Schiff base have been synthesized by refluxing the reaction mixture of hot ethanolic solution (30 ml) of 5-methyl-3-thiosemicarbazide (0.01 mol) and hot ethanolic solution (30 ml) of 5-nitroisatin (0.01 mol) for 2 hrs. The precipitate formed during reflux was filtered, washed with cold EtOH and recrystallized from hot EtOH (yield 80%, m.p. 579.8–580.3 K). The orange crystals were grown in an acetone-DMF (3:1) solution by slow evaporation at room temperature.

### Refinement

N-bound H atoms were located in a difference Fourier map and were refined freely. The remaining H atoms were positioned geometrically and refined using a riding model, with C—H = 0.95 Å for aromatic ring and C—H = 0.98 Å for methyl group, and with *U*_iso_(H) = 1.2*U*_eq_(C) and *U*_iso_(H) = 1.5*U*_eq_(C) for aromatic ring and methyl group, respectively. The highest residual electron density peak is located at 0.81 Å from C6 and the deepest hole is located at 0.36 Å from Sl.

### Crystal data

**Table:**

C_10_H_9_N_5_O_3_S	*F*(000) = 576
*M**_r_* = 279.28	*D*_x_ = 1.630 Mg m^−^^3^
Monoclinic, *P*2_1_/*c*	Melting point = 579.8–580.3 K
Hall symbol: -P 2ybc	Mo *K*α radiation, λ = 0.71073 Å
*a* = 4.6316 (4) Å	Cell parameters from 3870 reflections
*b* = 9.3157 (8) Å	θ = 2.7–30.1°
*c* = 26.458 (2) Å	µ = 0.30 mm^−^^1^
β = 94.485 (2)°	*T* = 100 K
*V* = 1138.09 (17) Å^3^	Needle, orange
*Z* = 4	0.36 × 0.12 × 0.07 mm

### Data collection

**Table:**

Bruker APEXII CCD diffractometer	2710 independent reflections
Radiation source: fine-focus sealed tube	2177 reflections with *I* > 2σ(*I*)
Graphite monochromator	*R*_int_ = 0.045
φ and ω scans	θ_max_ = 28.0°, θ_min_ = 2.3°
Absorption correction: multi-scan (*SADABS*; Bruker, 2005)	*h* = −6→6
*T*_min_ = 0.900, *T*_max_ = 0.979	*k* = −11→12
10734 measured reflections	*l* = −34→34

### Refinement

**Table:**

Refinement on *F*^2^	Primary atom site location: structure-invariant direct methods
Least-squares matrix: full	Secondary atom site location: difference Fourier map
*R*[*F*^2^ > 2σ(*F*^2^)] = 0.048	Hydrogen site location: inferred from neighbouring sites
*wR*(*F*^2^) = 0.102	H atoms treated by a mixture of independent and constrained refinement
*S* = 1.10	*w* = 1/[σ^2^(*F*_o_^2^) + (0.0189*P*)^2^ + 1.8143*P*] where *P* = (*F*_o_^2^ + 2*F*_c_^2^)/3
2710 reflections	(Δ/σ)_max_ = 0.001
185 parameters	Δρ_max_ = 0.40 e Å^−^^3^
0 restraints	Δρ_min_ = −0.29 e Å^−^^3^

### Special details

**Table:**

Geometry. All e.s.d.'s (except the e.s.d. in the dihedral angle between two l.s. planes) are estimated using the full covariance matrix. The cell e.s.d.'s are taken into account individually in the estimation of e.s.d.'s in distances, angles and torsion angles; correlations between e.s.d.'s in cell parameters are only used when they are defined by crystal symmetry. An approximate (isotropic) treatment of cell e.s.d.'s is used for estimating e.s.d.'s involving l.s. planes.
Refinement. Refinement of *F*^2^ against ALL reflections. The weighted *R*-factor *wR* and goodness of fit *S* are based on *F*^2^, conventional *R*-factors *R* are based on *F*, with *F* set to zero for negative *F*^2^. The threshold expression of *F*^2^ > σ(*F*^2^) is used only for calculating *R*-factors(gt) *etc*. and is not relevant to the choice of reflections for refinement. *R*-factors based on *F*^2^ are statistically about twice as large as those based on *F*, and *R*- factors based on ALL data will be even larger.

### Fractional atomic coordinates and isotropic or equivalent isotropic displacement parameters (Å^2^)

**Table:**

	*x*	*y*	*z*	*U*_iso_*/*U*_eq_	
S1	1.21633 (12)	0.51362 (6)	0.32848 (2)	0.01849 (15)	
O1	0.5410 (3)	0.21885 (18)	0.25632 (6)	0.0199 (4)	
O2	−0.1914 (4)	−0.2525 (2)	0.47157 (8)	0.0409 (5)	
O3	0.1838 (4)	−0.1372 (2)	0.50290 (7)	0.0304 (4)	
N1	0.2084 (4)	0.0484 (2)	0.27784 (8)	0.0187 (4)	
N2	0.6885 (4)	0.2193 (2)	0.36918 (7)	0.0159 (4)	
N3	0.8430 (4)	0.3086 (2)	0.34179 (7)	0.0166 (4)	
N4	1.0960 (4)	0.3840 (2)	0.41442 (7)	0.0189 (4)	
N5	0.0142 (5)	−0.1717 (2)	0.46698 (8)	0.0242 (5)	
C1	0.1322 (5)	−0.0181 (2)	0.32196 (8)	0.0167 (5)	
C2	−0.0740 (5)	−0.1225 (3)	0.32723 (9)	0.0198 (5)	
H2A	−0.1871	−0.1592	0.2986	0.024*	
C3	−0.1108 (5)	−0.1721 (3)	0.37559 (9)	0.0212 (5)	
H3A	−0.2502	−0.2444	0.3807	0.025*	
C4	0.0580 (5)	−0.1154 (3)	0.41667 (9)	0.0198 (5)	
C5	0.2677 (5)	−0.0110 (3)	0.41212 (9)	0.0185 (5)	
H5A	0.3813	0.0249	0.4408	0.022*	
C6	0.3033 (5)	0.0383 (2)	0.36375 (8)	0.0166 (5)	
C7	0.4952 (5)	0.1430 (2)	0.34383 (8)	0.0155 (4)	
C8	0.4257 (5)	0.1451 (2)	0.28722 (8)	0.0166 (5)	
C9	1.0477 (5)	0.3984 (2)	0.36484 (8)	0.0164 (5)	
C10	1.3117 (5)	0.4679 (3)	0.44437 (9)	0.0257 (6)	
H10A	1.2463	0.4842	0.4782	0.039*	
H10B	1.3383	0.5604	0.4277	0.039*	
H10C	1.4959	0.4157	0.4473	0.039*	
H1N3	0.803 (6)	0.317 (3)	0.3104 (11)	0.020 (7)*	
H1N4	1.013 (6)	0.318 (3)	0.4291 (10)	0.021 (7)*	
H1N1	0.123 (6)	0.039 (3)	0.2501 (11)	0.024 (7)*	

### Atomic displacement parameters (Å^2^)

**Table:**

	*U*^11^	*U*^22^	*U*^33^	*U*^12^	*U*^13^	*U*^23^
S1	0.0212 (3)	0.0194 (3)	0.0137 (3)	0.0002 (2)	−0.0055 (2)	0.0021 (2)
O1	0.0248 (8)	0.0222 (9)	0.0117 (8)	0.0021 (7)	−0.0052 (7)	−0.0004 (7)
O2	0.0460 (12)	0.0433 (12)	0.0332 (11)	−0.0198 (10)	0.0027 (9)	0.0087 (10)
O3	0.0335 (10)	0.0384 (11)	0.0183 (9)	−0.0006 (8)	−0.0046 (8)	0.0046 (8)
N1	0.0225 (10)	0.0215 (10)	0.0107 (10)	0.0014 (8)	−0.0081 (8)	−0.0037 (8)
N2	0.0173 (9)	0.0160 (9)	0.0135 (9)	0.0022 (7)	−0.0039 (7)	−0.0008 (8)
N3	0.0211 (10)	0.0193 (10)	0.0082 (9)	0.0007 (8)	−0.0062 (7)	0.0012 (8)
N4	0.0221 (10)	0.0222 (11)	0.0111 (10)	−0.0047 (8)	−0.0055 (8)	−0.0006 (8)
N5	0.0274 (11)	0.0227 (11)	0.0224 (11)	0.0017 (9)	0.0013 (9)	0.0036 (9)
C1	0.0173 (10)	0.0165 (11)	0.0153 (11)	0.0060 (9)	−0.0047 (8)	−0.0041 (9)
C2	0.0190 (11)	0.0189 (12)	0.0203 (12)	0.0019 (9)	−0.0066 (9)	−0.0068 (10)
C3	0.0216 (11)	0.0155 (11)	0.0258 (13)	0.0013 (9)	−0.0015 (10)	−0.0016 (10)
C4	0.0226 (11)	0.0186 (12)	0.0177 (12)	0.0045 (9)	−0.0012 (9)	0.0010 (9)
C5	0.0183 (10)	0.0188 (12)	0.0176 (11)	0.0039 (9)	−0.0037 (9)	−0.0020 (9)
C6	0.0165 (10)	0.0170 (11)	0.0155 (11)	0.0037 (8)	−0.0041 (9)	−0.0034 (9)
C7	0.0175 (10)	0.0170 (11)	0.0111 (11)	0.0053 (8)	−0.0043 (8)	−0.0016 (9)
C8	0.0190 (11)	0.0182 (11)	0.0113 (11)	0.0049 (9)	−0.0063 (8)	−0.0032 (9)
C9	0.0174 (10)	0.0156 (11)	0.0152 (11)	0.0047 (9)	−0.0048 (9)	−0.0036 (9)
C10	0.0283 (13)	0.0315 (14)	0.0157 (12)	−0.0081 (11)	−0.0086 (10)	−0.0036 (11)

### Geometric parameters (Å, º)

**Table:**

S1—C9	1.675 (2)	C1—C2	1.378 (3)
O1—C8	1.222 (3)	C1—C6	1.410 (3)
O2—N5	1.227 (3)	C2—C3	1.383 (3)
O3—N5	1.227 (3)	C2—H2A	0.9500
N1—C8	1.359 (3)	C3—C4	1.392 (3)
N1—C1	1.391 (3)	C3—H3A	0.9500
N1—H1N1	0.81 (3)	C4—C5	1.387 (3)
N2—C7	1.289 (3)	C5—C6	1.382 (3)
N2—N3	1.346 (3)	C5—H5A	0.9500
N3—C9	1.371 (3)	C6—C7	1.446 (3)
N3—H1N3	0.84 (3)	C7—C8	1.507 (3)
N4—C9	1.320 (3)	C10—H10A	0.9800
N4—C10	1.454 (3)	C10—H10B	0.9800
N4—H1N4	0.84 (3)	C10—H10C	0.9800
N5—C4	1.460 (3)		
			
C8—N1—C1	112.00 (19)	C5—C4—N5	118.6 (2)
C8—N1—H1N1	123 (2)	C3—C4—N5	117.8 (2)
C1—N1—H1N1	125 (2)	C6—C5—C4	116.7 (2)
C7—N2—N3	115.95 (19)	C6—C5—H5A	121.7
N2—N3—C9	121.06 (19)	C4—C5—H5A	121.7
N2—N3—H1N3	120.3 (18)	C5—C6—C1	120.1 (2)
C9—N3—H1N3	118.3 (18)	C5—C6—C7	133.1 (2)
C9—N4—C10	122.9 (2)	C1—C6—C7	106.81 (19)
C9—N4—H1N4	119.1 (19)	N2—C7—C6	127.1 (2)
C10—N4—H1N4	117.7 (19)	N2—C7—C8	126.5 (2)
O2—N5—O3	122.6 (2)	C6—C7—C8	106.34 (19)
O2—N5—C4	118.3 (2)	O1—C8—N1	127.4 (2)
O3—N5—C4	119.1 (2)	O1—C8—C7	127.0 (2)
C2—C1—N1	128.5 (2)	N1—C8—C7	105.67 (19)
C2—C1—C6	122.4 (2)	N4—C9—N3	116.0 (2)
N1—C1—C6	109.2 (2)	N4—C9—S1	125.82 (18)
C1—C2—C3	117.8 (2)	N3—C9—S1	118.22 (17)
C1—C2—H2A	121.1	N4—C10—H10A	109.5
C3—C2—H2A	121.1	N4—C10—H10B	109.5
C2—C3—C4	119.5 (2)	H10A—C10—H10B	109.5
C2—C3—H3A	120.2	N4—C10—H10C	109.5
C4—C3—H3A	120.2	H10A—C10—H10C	109.5
C5—C4—C3	123.6 (2)	H10B—C10—H10C	109.5
			
C7—N2—N3—C9	177.82 (19)	C2—C1—C6—C7	179.3 (2)
C8—N1—C1—C2	−178.9 (2)	N1—C1—C6—C7	−0.7 (2)
C8—N1—C1—C6	1.1 (3)	N3—N2—C7—C6	179.3 (2)
N1—C1—C2—C3	−179.9 (2)	N3—N2—C7—C8	−0.4 (3)
C6—C1—C2—C3	0.0 (3)	C5—C6—C7—N2	−0.5 (4)
C1—C2—C3—C4	0.3 (3)	C1—C6—C7—N2	−179.7 (2)
C2—C3—C4—C5	−0.7 (4)	C5—C6—C7—C8	179.3 (2)
C2—C3—C4—N5	−179.4 (2)	C1—C6—C7—C8	0.1 (2)
O2—N5—C4—C5	172.4 (2)	C1—N1—C8—O1	179.1 (2)
O3—N5—C4—C5	−7.9 (3)	C1—N1—C8—C7	−1.0 (2)
O2—N5—C4—C3	−8.9 (3)	N2—C7—C8—O1	0.2 (4)
O3—N5—C4—C3	170.8 (2)	C6—C7—C8—O1	−179.6 (2)
C3—C4—C5—C6	0.8 (3)	N2—C7—C8—N1	−179.7 (2)
N5—C4—C5—C6	179.5 (2)	C6—C7—C8—N1	0.5 (2)
C4—C5—C6—C1	−0.5 (3)	C10—N4—C9—N3	178.2 (2)
C4—C5—C6—C7	−179.5 (2)	C10—N4—C9—S1	−0.9 (3)
C2—C1—C6—C5	0.1 (3)	N2—N3—C9—N4	4.5 (3)
N1—C1—C6—C5	180.0 (2)	N2—N3—C9—S1	−176.25 (16)

### Hydrogen-bond geometry (Å, º)

**Table:**

*D*—H···*A*	*D*—H	H···*A*	*D*···*A*	*D*—H···*A*
N3—H1*N*3···O1	0.84 (3)	2.02 (3)	2.697 (2)	137 (3)
N1—H1*N*1···S1^i^	0.81 (3)	2.52 (3)	3.320 (2)	171 (3)
C2—H2*A*···O1^ii^	0.95	2.39	3.317 (3)	165
C10—H10*A*···O2^iii^	0.98	2.56	3.079 (3)	113

Symmetry codes: (i) −*x*+1, *y*−1/2, −*z*+1/2; (ii) −*x*, *y*−1/2, −*z*+1/2; (iii) −*x*+1, −*y*, −*z*+1.
